# Cost–Benefit Analysis of a Mass Vaccination Strategy to Control Brucellosis in Sheep and Goats in Northern Iraq

**DOI:** 10.3390/vaccines9080878

**Published:** 2021-08-08

**Authors:** Ali Al Hamada, Mieghan Bruce, Anne Barnes, Ihab Habib, Ian D. Robertson

**Affiliations:** 1 School of Veterinary Medicine, Murdoch University, Perth 6150, Australia; Gaddan76@gmail.com (A.A.H.); Mieghan.Bruce@murdoch.edu.au (M.B.); i.robertson@murdoch.edu.au (I.D.R.); 2 College of Veterinary Medicine, University of Mosul, Mosul 41002, Iraq; 3 Centre for Animal Production and Health, Food Futures Institute, Murdoch University, Perth 6150, Australia; 4 Department of Veterinary Medicine, College of Food and Agriculture, United Arab Emirates University (UAEU), Al Ain P.O. Box 1555, United Arab Emirates; 5 College of Veterinary Medicine, Huazhong Agricultural University, Wuhan 430070, China

**Keywords:** brucellosis, economic analysis, vaccination, small ruminants, Iraq

## Abstract

Brucellosis is a major economic and production-limiting disease for livestock owners and the community in Iraq. A cost–benefit analysis was conducted to evaluate the impact of an expanded annual mass vaccination programme of sheep and goats that involves all female and male sheep and goats over the age of 3 months with Rev. 1 vaccine. The proposed expanded vaccination programme was compared to the current annual vaccination program, which involved only vaccinating female sheep and goats between the ages of 3 and 6 months of age with Rev. 1. The cost-benefit analysis model was developed utilizing data collected in Dohuk Governorate, northern Iraq. The seroprevalence in small ruminants (using Rose Bengal test and ELISA in series) was predicted to decrease from 9.22% to 0.73% after 20 years of implementing the proposed annual mass vaccination program. The net present value of the mass vaccination program was estimated to be US$ 10,564,828 (95% Confidence Interval (CI): −16,203,454 to 37,049,245), the benefit–cost ratio was estimated to be 4.25 (95% CI: −2.71 to 11.22), and the internal rate of return was 91.38% (95% CI:11.71 to 190.62%). The proposed vaccination strategy was predicted to decrease the overall financial loss caused by brucellosis from 1.75 to 0.55 US$ per adult female animal. The results of this economic analysis highlight the benefit of implementing an annual mass vaccination program of small ruminants with Rev. 1 vaccine to reduce the prevalence of brucellosis in northern Iraq.

## 1. Introduction

Brucellosis is a common zoonotic disease in many countries resulting in production losses in livestock and febrile disease in humans [1,2]. Control has proved elusive in many countries, particularly where *Brucella melitensis*, the more pathogenic species for humans and small ruminants (sheep and goats), dominates [3,4]. Although *B. melitensis* has been eradicated from small ruminant flocks in most industrialised countries, it remains a significant burden on small ruminants and human health in the Mediterranean region, the Middle East, Central and Southeast Asia (including India and China), sub-Saharan Africa, and certain areas in Latin America [5]. The economic impact of brucellosis varies depending upon the prevalence and management and husbandry systems adopted, along with the local veterinary and medical services’ expertise, facilities, and capacity [2]. In small ruminants, the disease can cause significant economic loss from abortions, neonatal deaths, reduced fertility and decreased milk production [6]. In addition, trade restrictions on livestock and products from infected areas further the impact of the disease on a community [2].

Brucellosis is endemic in small ruminants in most countries of the Mediterranean Basin and the Middle East [5]. In Iraq, incidences of brucellosis in humans of between 52.3 cases per 100,000 person-years in a rural area to 268.8 cases per 100,000 person-years in a semi-rural area in the Basra region (south of Iraq) have been recorded [7], with infection primarily arising through the handling of infected animals or consumption of unpasteurized milk or other dairy products [8,9]. Clinical symptoms in humans are generally nonspecific, leading to the potential for under-reporting of the disease [10]. As human infection primarily arises from infected animals or their products, controlling the disease in livestock is critical to reducing the incidence in humans [11]. The primary control strategy adopted in animals is vaccination, in conjunction with surveillance, quarantine, culling of infected animals, and adoption of good biosecurity, husbandry, and management systems [12].

Eradication of brucellosis in small ruminants remains challenging in many developing countries [6]. In many developing countries, including Iraq, brucellosis is an endemic disease in small ruminants, resulting in significant impacts on livestock productivity and the livelihood of people [12,13,14]. The lack of effective control programs in Iraq exacerbates the disease’s impact, and there has been a call for adopting a ‘One Health’ approach to solve this endemic problem [13,14]. In northern Iraq, small ruminants and their products are vital to the economy of the Kurdistan Region of Iraq (KRI) and improve the quality of life for the local community [12]. Of the many endemic diseases that affect sheep and goats in Dohuk Governorate, located in the KRI, *B. melitensis* continues to pose a threat to livestock productivity, as well as to public health [14]. With little change in the prevalence of brucellosis in KRI or the current vaccination programme (vaccinating young animals, between three and six months of age, only), brucellosis is considered at endemic equilibrium, which calls for a change in the vaccination strategy. Where brucellosis is endemic, with a high prevalence, and where flocks are managed extensively, such as northern Iraq, mass vaccination is considered the best method, and frequently the only reasonable strategy, to apply in such settings [12,13]. Hence, the current study aimed to evaluate the economic value of undertaking an expanded annual mass vaccination programme targeting all female and male sheep and goats older than three months with the Rev. 1 live vaccine. This study compares the alternative vaccination programme against the current programme (status quo) vaccinating young animals only.

## 2. Materials and Methods

### 2.1. Study Scope and Context

In this study, a cost–benefit analysis was undertaken to compare an annual mass vaccination control programme involving vaccination of all female and male sheep and goats older than three months of age over a 20-year period. The time frame (20 years) simulates a long-term vaccination strategy to decrease the prevalence of brucellosis in the study setting to a level (~5%) where a test-and-slaughter control programme could be applied [15]. The study setting is Dohuk Governorate, located in the KRI of northern Iraq and bordering Syria and Turkey. The governorate is populated by approximately 1.2 million people and contains about 1 million sheep and goats [16]. 

### 2.2. Economic Model

#### 2.2.1. Data Sources and Model Input Parameters

Inputs for the economic model (Table 1) were sourced from: (i) a prospective cohort study conducted in Dohuk Governorate, where a sample of pregnant sheep flocks was followed over time to evaluate brucellosis incidence, seroconversion, and outcome of pregnancy [17]; (ii) a cross-sectional study conducted in Dohuk, which involved a serosurvey of sheep and goats from 72 farms [13]; and (iii) a questionnaire conducted on the same farms to gather information on production and prices. Where no data were available in the study area, input parameters were extracted from published literature. A total of 242,405 sheep and goats were recorded by the annual report from Dohuk Veterinary Hospital in 2015 to be owned by the surveyed farmers in that study [13], of which 70.7% (171,336) were adult females (over one year of age), 3.3% (8064) were adult males older than one year, and 26% (63,005) were lambs or kids less than one year of age. 

The cost–benefit model in this study was developed in Microsoft Excel 2010 with the add-on package @Risk (version 7.5, Palisade Corporation, New York, NY, USA.). A Monte Carlo simulation with Latin hypercube sampling was carried out to simulate the distribution depicting some of the model variables (Table 1) and to account for variability and uncertainty associated with such variables. The PERT distribution was used with data from the literature to specify the minimum, mode (most likely), and maximum values. The model was run for 10,000 iterations. The model inputs and parameters are summarised in Table 1. A discount rate of 6.0% was applied as the reported discount rate used by the central bank of Iraq [18], and a sensitivity analysis using 5% and 10% was conducted. 

The average price of a lamb or kid was estimated at US$50 (data from questionnaire gathered at the study site). The cost of the vaccine was included at US$ 0.10 per head in this study (personal communication of Dohuk Veterinary Hospital, Dohuk). The true prevalence was calculated from the apparent prevalence (AP) of brucellosis in female sheep and goats using RBT and ELISA in series [13], together with published values about test characteristics (sensitivity (Se) and specificity (Sp)) (Table 1) [19,20]. The attributable risk of pregnancy loss in new (incidence) cases and in existing (prevalence) cases in sheep and goats was 25% and 18%, respectively [17]. 

#### 2.2.2. Model Assumptions and Simplifications

Several simplifying assumptions were made here to render the modeling and analysis more tractable while retaining adequate accuracy to ensure meaningful results:

Implementation cost: It was assumed that 24 vaccinator teams would be required, with each team comprising one veterinarian, two veterinary nurses, and one driver. These teams would work for the Veterinary Medical Centres in the districts and sub-districts for 30 days each year to ensure 100% vaccination coverage would be achieved (personal experience, as recommended by the Director of Dohuk Veterinary Hospital). It was assumed that the teams could vaccinate between 1000 and 3000 small ruminants a day (average 1400). The direct costs for the vaccination program included the salaries of the vaccination team members, transport costs of the vaccine, transportation of the team members to the vaccination sites (flocks), consumables, and materials to keep the vaccines chilled (cold chain). Based on local setting (informed by the first author in this study), US$20 per day was estimated for general consumables, meetings, and transport (including ice for packaging vaccines, fuel, syringes, personal protective equipment and waste disposal, expenses for sub-district meetings and administration) per team. Thus, total implementation cost (based on the above description) = US$ 20 × 30 (days-period of vaccination programme) × 24 (numbers of teams) = US$ 14,400.

Budget for salaries: Was allocated as US$ 3200 salary per team per month. Thus, total budgeted salaries = 24 (numbers of teams) × 3200 = US$ 76,800

Budget for vaccine allocation: It was estimated that the *Brucella* vaccine would cost US$ 0.10 per dose, and the total number of small ruminants requiring vaccination was 1,000,000 (according to the most recent annual report of Dohuk Veterinary Hospital). Thus, the total estimated budget for vaccine cost = US$ 100,000.

The total costs of the mass vaccination control program per year of the program were estimated at US$ 191,200 = US$ 14,400 (implementation) + US$ 76,800 (salaries) + US$ 100,000 (vaccine allocation). 

#### 2.2.3. Productivity and Reproduction Impacts

The Awassi is the leading sheep breed in Iraq [21], and data on their fertility and milk production were sourced from several existing reports (Table 1). As there is a lack of available data regarding the productivity of local goats in Iraq, we used the same values for sheep and goats (Table 1). The economic impact of the disease in sheep and goats was assessed based on its influence only on milk production (per liter) and abortions, as these are considered to be among the most relevant impacts arising from the disease [6]. This study assessed the impact of brucellosis on reproduction and productivity according to the formulas listed below [11]. Cases of mastitis, hygromas, orchitis, and epididymitis were not factored into this analysis as little data were available regarding these features of the disease in sheep or goats in Iraq, and given the fact that the significant impact of the disease is attributed to abortion, infertility and reduced milk yield [11].

Production losses = **A** + **M**

where **A:** is the cost of an abortion [A = (Number of prevalent infected females * attributable risk (for prevalent cases) * Fertility rate * Average price of one lamb or kid (US$)) + (Number of newly infected females * attributable risk (for new cases) * Fertility rate * Average price of one lamb or kid (US$))]. **M:** is the cost of reduced milk production [M = (Number of prevalent infected females * attributable risk (in existing (prevalence) cases) * Fertility rate * Milk production per adult female per year * Average price of milk per liter (US$)) + (Number of newly infected females * attributable risk (for new cases) * Fertility rate * Milk production per adult female per year * Average price of milk per liter (US$))].

Annual loss per seropositive animal = **T** ÷ **I**

where **T**: is the total loss due to brucellosis in sheep and goats in Dohuk Governorate. **I**: is the number of seropositive animals (number of infected animals).

#### 2.2.4. Strategy and Control of the Disease

Cost–benefit analysis is a method that is commonly used to determine whether the benefit from a control programme exceeds the costs of conducting it [22]. The net present value (NPV), benefit–cost ratio (BCR), and internal rate of return (I.R.R) were calculated [22]. The median values and their 95% confidence intervals were calculated for NPV, BCR, and I.R.R using@ Risk software (version 7.5, Palisade Corporation, New York, NY, USA).

This study assumed that 60% vaccination coverage of all female and male sheep and goats older than three months was achieved using a standard dose of Rev. 1 administered in the conjunctiva [15]. It was assumed that the number of female sheep and goats kept each year for replacement purposes was equal to the number of female sheep and goats that died or were culled each year, i.e., the overall population size was stable. It was also assumed that brucellosis in Dohuk Governorate was at an endemic equilibrium at the start of the study where the number of newly infected animals produced by one infected animal (sheep or goat) during its infectious period (effective reproduction number, R_e_) was 1. The calculation of the number of new cases of brucellosis in each year was derived from the formula as follows [23]:

R_e_ = R_0_ × s

where R_e_ is the effective reproduction number, which is the average number of secondary cases that result from an infectious individual in a particular population. R_0_ is the basic reproduction ratio (the mean number of secondary cases arising from one infectious individual in an entirely susceptible population). s is the proportion of susceptible animals in the total population.

The economic value of the mass vaccination programme was calculated as the financial savings arising from the reduced number of infected female sheep and goats (increased milk production and reduced abortions) and the extra costs associated with a mass vaccination program compared with the current vaccination programme (vaccination of young sheep and goats only).

#### 2.2.5. Sensitivity Analysis

A sensitivity analysis was conducted (in @Risk), using the Spearman rank correlation coefficient (*r*), to determine the impact of variability and uncertainty for the input parameters on the predicted model output. A sensitivity analysis was carried out to assess the effect of the protection rate from vaccination on the NPV, BCR, and I.R.R from the mass vaccination strategy. The protection rate was based on both the efficacy of the vaccine and the vaccination coverage achieved.

## 3. Results

**Figure 1:**
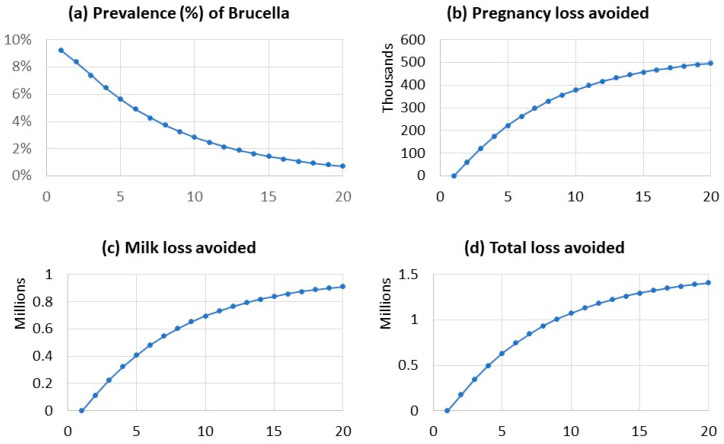
Estimated decline in the prevalence of Brucella (**a**) when implementing a mass vaccination programme over twenty years (horizontal axis). The figure depicts the avoided pregnancy loss (**b**), avoided milk loss (**c**), and the predicted total loss avoided (**d**) over twenty years of application of the proposed mass vaccination.

The prevalence of brucellosis in sheep and goats in Dohuk was predicted to decline over the 20-year period from 9.22% to 0.73% (Figure 1a). The analysis predicted that, after adopting a mass vaccination control program for 20 years, the annual losses from pregnancy loss and decreased milk production had decreased to US$115,299 (US$0.16 per adult female). The total economic impact of brucellosis per year in sheep and goats at the start of the control program in Dohuk Governorate (2015) was estimated to be US$1,524,151 (US$2.16 per adult female).

The results of the cost–benefit analysis are presented in Table 2 and Table 3. The median total benefit in present day dollars was estimated at US$ 13,813,524 (95% CI:−12,964,774–40,290,145). For the mass vaccination control programme compared to the existing control programme using 4% interest rate, the median NPV was US$ 10,564,828 (95% CI: −16,203,454–37,049,245), and the median BCR was 4.255 (95% CI: −2.71–11.22). The median of IRR was 91.38% (95% CI: 11.7–190.6%) (Table 3). The median total costs of the mass vaccination program over the 20-year period were estimated at US$ $ 3,241,685 (95% CI: 2,971,912–3,515,547) (Table 3). 

**Figure 2:**
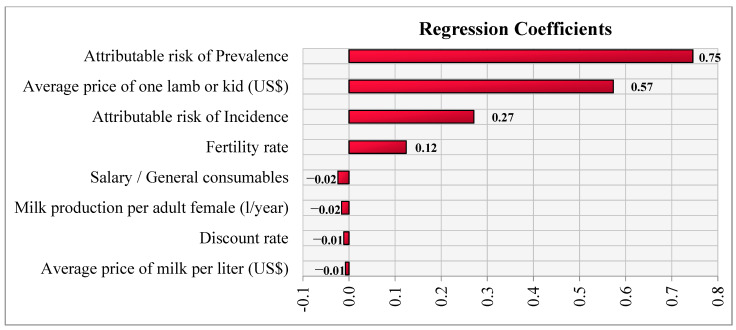
Regression coefficients of the sensitivity analysis for net present value (NPV) of the mass vaccination control programme.

In the sensitivity analysis (Figure 2), the attributable risk of pregnancy loss (prevalent cases) (regression coefficient = 0.75) had the most considerable positive effect on the out-come, followed by the average price of one lamb or kid (0.57), and the attributable risk of incidence loss of pregnancy (regression coefficient = 0.27). All other factors had minimal impact on the control program (low coefficients) (Figure 2).

## 4. Discussion

The current study was oriented on analysis of the economic losses occurring due to brucellosis in small ruminants in Iraq. This analysis focused on impacts arising from loss of pregnancy and reduced milk yield, as these have been reported to be among the most significant outcomes of infection [11,27]. In this study, we populated the cost–benefit analysis model based on integrated data from our previous research on brucellosis in small ruminants in the study area; key model parameters were informed based on a recent cohort study [17] and a former cross-sectional serological survey carried out by our group in Dohuk [13].

This study found that the proposed mass vaccination programme had an NPV greater than zero and a BCR greater than one, indicating that the programme was economically beneficial. It is likely that the benefits in this study and the corresponding BCR and NPV would have been higher if data were included in the model on all potential losses in animals along with the impact of the disease in humans. The present cost–benefit analysis predicted that the losses decreased from US$2.16 to US$0.16 per adult female over the 20-year period, and the seroprevalence decreased from 9.22% to 0.73%. Compared to our study, another study in the Kurdistan region in Iraq found that the loss in animals was decreased over ten years from US$2.56 to US$0.76 per adult female based on seroprevalence 4.9% [12]. In addition, the IRR in our study was estimated to be 74%, which was slightly higher than another recent study conducted in the Kurdistan region of Iraq, where an IRR of 67.9% was reported [12]. Although the losses resulting from infection with *B. melitensis* have not been investigated widely in developing countries, some studies have been undertaken in middle-income countries. In Malaysia, based on a reported seroprevalence of 2.9% using the complement fixation test, it was estimated that annually the economic impact of caprine brucellosis was US$ 50,391.13 in 15 herds containing a total of 12,499 goats. This resulted in an average loss of US$ 162.55 for the 310 infected animals [28]. In another study in India, the annual economic losses per animal were estimated at US$ 21.58 and US$ 38.80 per goat [29]. 

In this study, the proposed expanded vaccination program in Dohuk Governorate was estimated to cost in PV US$9.1 million over the 20-year period. Although the infection was predicted to remain after 20 years of vaccination, the true prevalence decreased to 0.73%. Others have similarly shown that mass vaccination programs over ten years can significantly reduce the prevalence and transmission of brucellosis between animals [30]. In another study, mass vaccination with the Rev. 1 vaccine over four and a half years reduced the prevalence of *B. melitensis* in Kuwait’s small ruminant population from 5.8% in 1993 to 2.02% in 1997 [31]. Although vaccination does not eliminate infection, the model predicted a significant reduction in prevalence after 20 years, at which time it might be possible to evaluate adopting eradication programs, such as test and slaughter programs [32]. Nevertheless, implementing a test and culling programme will be challenging unless a formal compensation system is developed for the culled animals. Raising awareness of the farming community regarding biosecurity and preventative measures is critical for any disease control program and should be a vital component of any official disease control program in the north of Iraq [13]. 

The results of this and other studies indicate that mass vaccination with Rev. 1 is a crucial strategy to reduce the prevalence of brucellosis arising from infection with *B. melitensis* in small ruminants, mainly when the prevalence is initially high [33,34]. Our scenario assumed that the disease had reached an endemic equilibrium with the current vaccination programme involving the vaccination of sheep and goats 3 to 6 months of age. Brucellosis-positive animals were still present in Dohuk Governorate, presumably due to a lack of collaboration by farmers regarding vaccination time, and absence of communication of the presence of young animals eligible for Rev. 1 vaccination. In addition, the illegal movement of animals of unknown disease status from Syria and Iran to Iraq and specifically to the KRI and the uncontrolled movement of domestic animals within the region are likely responsible for the wide dissemination of brucellosis throughout northern Iraq. 

Others have similarly shown the impact of vaccination with Rev. 1 on reducing disease prevalence in sheep and goats in countries where the disease is endemic, including Mexico [35] and India [36]. In a study in Greece, vaccination of sheep and goats with Rev. 1 vaccine administered subcutaneously was shown to decrease abortion in small ruminants and the incidence of brucellosis in humans [37]. Abortion is the primary clinical outcome of brucellosis infection in sheep and goats and typically occurs in the last two months of pregnancy [38]. Not surprisingly, the abortion rate had the most significant influence on the sensitivity analysis in this study. The mass vaccination scenario proposed should reduce the number of abortions, reducing potential environmental contamination [36]. Although Rev. 1 vaccine induces effective immunity against brucellosis in sheep and goats [39], it has disadvantages, including the potential to result in abortions when administered to pregnant females due to the live vaccine containing mutants with residual virulence [40]. Therefore, it is recommended that female sheep and goats are vaccinated before the breeding season commences and at milking season.

Although annual mass vaccination was predicted not to eliminate *Brucella* infection over 20 years, the program predicted a sizeable reduction in the prevalence, allowing for implementation of a test and slaughter programme, similar to that used in other countries [2]. Furthermore, because infected animals and their products are the primary sources of human infections, control of brucellosis in animals is an essential step to mitigating the disease in humans [11].

The findings of this study have to be seen in the light of some limitations. This study only focused on vaccination in small ruminants; however, brucellosis can be transmitted between small ruminants and cattle, particularly in co-grazing situations, which could be common in some regions in Iraq [41,42], and this is an area open for investigation in the context of Iraq in future research. Some assumptions and extrapolations of other studies were required due to a lack of local data. Future studies should be undertaken to collect local data to ensure the model is as accurate as possible for the situation in Dohuk and Iraq. Inclusion in the economic modelling of other costs and benefits associated with milk production and fertility rate due to brucellosis with brucellosis control will also help generate a more accurate model.

It is recommended that an expanded vaccination program is implemented in Iraq in conjunction with a program to increase the awareness of the local farmers about the disease, its impact, and how it is spread through implementing educational campaigns by the regional Department of Veterinary Services. It is critical to focus on vaccination and concurrently build a robust disease surveillance system involving monitoring all abortion cases by the provincial veterinary system to prevent the spread of disease or reinfection. In addition, developing a solid quarantine system and control on the illegal movement of animals from neighbouring countries, especially Syria and Iran, are essential for the ongoing control of brucellosis (and other diseases) in Dohuk Governorate. Controlling brucellosis in small ruminants is essential to reduce the economic impact of the disease in sheep and goats and lessen the infection burden in humans [34]. 

## 5. Conclusions

Adoption of a mass vaccination programme involving vaccination of all females and males older than three months of age with Rev. 1 in Dohuk Governorate, Iraq, was predicted to lead to a reduction in the prevalence of brucellosis from 36 % to 6.6% after 20 years of implementation. The program was shown to be economically advantageous, with a median NPV of US$ 10,564,828 and a median BCR of 4.25. It is concluded that the Iraqi government should plan and implement an evidence-based vaccination program in Dohuk, and other areas of Iraq, for the control and future eradication of brucellosis in sheep and goats based on initially reducing the prevalence through this vaccination campaign.

## Figures and Tables

**Table 1:** Input parameters included in the cost–benefit analysis model comparing two different vaccination strategies to control brucellosis in sheep and goats in Dohuk, northern Iraq.

Parameters	Description	Value	Reference/Source
Annual milk production	litre per adult female (ewe/doe) per year	Pert distribution (Min = 60; Mode = 109; Max = 134)	Pacinovski, et al. [24]
Fertility rate	Per year, for adult female %	Pert distribution (Min = 0.76; Mode = 0.85; Max = 0.95)	Galal, et al. [25]
Average price of milk *	Per kg	Pert distribution (Min = 0.6317; Mode = 0.8421; Max = 1.0528)	Questionnaire data
Average price of a lamb or kid	Per a lamb or kid	Pert distribution (Min = 25; Mode = 50; Max = 75)	Questionnaire data
Percentage of adult females	Out of the total population	70.7%	Questionnaire data
Percentage of lambs/kids	Out of the total population	26%	Questionnaire data
Attributable risk	For new (incidence) cases	Pert distribution (Min = 0.05; Mode = 0.25; Max = 0.45)	AlHamada, et al. [17]
Attributable risk	For existing (prevalence) cases	Pert distribution (Min = 0; Mode = 0.18; Max = 0.34)	AlHamada, et al. [17]
TP	True prevalence (TP) = $\frac{\left(\mathrm{AP}\;+\;\mathrm{Sp}\;-1\right)\;}{\mathrm{Se}\;+\;\mathrm{Sp}\;-1}$	9.22%	Calculated
AP	Apparent prevalence (AP)	8.33%	Alhamada, et al. [13]
Se	Sensitivity (series testing)	90.22%	EFSA [20] and Nielsen, et al. [19]
Sp	Specificity (series testing)	99.99%	EFSA [20] and Nielsen, et al. [19]
s	Proportion of susceptible animals [t = 0]	0.46	Calculation (s = 1 − TP − Pr)
Pr	Proportion of protected animals [t = 0]	0.45	Calculation (Pr = Vc × Ve)
Vc	Vaccination coverage	60%	Directorate of Dohuk Veterinary Hospital
Ve	Vaccine efficacy	75%	Benkirane, et al. [26]
N	Number of adult female sheep and goats in Dohuk Governorate	706,800	Total number of animals × percentage of adult females
P	Number of protected females in Dohuk Governorate (year 0)	299,115	Calculated (P = (N × Pr))
Re	Effective reproduction number	1	assumption of endemic equilibrium
R0	Basic reproduction numberR_0_ = Re ÷ s	2.18	Hegazy, et al. [23]
D	Duration of overall managed breeding before culling	Five years	Calculation (1 ÷ u)
U	Replacement sheep per year (Cull rate)	20%	Director of the vet. Services in Dohuk city
beta	Transmission coefficient	6.18 × 10^−7^	Calculated beta = R_e_ ÷ (N × D × s)
Rev. 1 vaccine	Price per dose	US$0.10	Directorate of Dohuk Veterinary in Dohuk city

* Prices expressed in US$ (United States of America dollar, with US$1 = 1190 Iraqi Dinar) (https://www.mataf.net/ (accessed on 2 May 2021)).

**Table 2:** Summary of the results of the benefit–cost analysis comparing an expanded vaccination control programme compared with a continuation of the current control programme.

Years	Future Benefits	Future Costs	Future Value	PV of Benefits	PV of Costs	NPV
1	$0	$191,200	−$191,200	$0	$179,728	−$179,728
2	$176,770	$191,200	−$14,430	$156,194	$168,944	−$12,750
3	$347,854	$191,200	$156,654	$288,922	$158,808	$130,114
4	$499,649	$191,200	$308,449	$390,101	$149,279	$240,821
5	$632,108	$191,200	$440,908	$463,907	$140,322	$323,584
6	$747,260	$191,200	$556,060	$515,512	$131,903	$383,609
7	$847,307	$191,200	$656,107	$549,460	$123,989	$425,471
8	$934,257	$191,200	$743,057	$569,494	$116,550	$452,944
9	$1,009,865	$191,200	$818,665	$578,647	$109,557	$469,091
10	$1,075,649	$191,200	$884,449	$579,361	$102,983	$476,377
11	$1,132,914	$191,200	$941,714	$573,592	$96,804	$476,788
12	$1,182,789	$191,200	$991,589	$562,913	$90,996	$471,917
13	$1,226,245	$191,200	$1,035,045	$548,579	$85,536	$463,043
14	$1,264,123	$191,200	$1,072,923	$531,593	$80,404	$451,189
15	$1,297,150	$191,200	$1,105,950	$512,753	$75,580	$437,173
16	$1,325,955	$191,200	$1,134,755	$492,691	$71,045	$421,646
17	$1,351,084	$191,200	$1,159,884	$471,906	$66,782	$405,124
18	$1,373,011	$191,200	$1,181,811	$450,791	$62,775	$388,016
19	$1,392,148	$191,200	$1,200,948	$429,650	$59,009	$370,641
20	$1,408,853	$191,200	$1,217,653	$408,717	$55,468	$353,249
Total	$19,224,992	$3,824,000	$15,400,992	$9,074,783	$2,126,463	$6,948,320

NPV: net present value; PV: present value.

**Table 3:** Summary of a cost–benefit analysis of a mass vaccination program applied for twenty years to control brucellosis in small ruminants in Dohuk Governorate, Iraq.

	Benefits (Median and 95% CI)
PV Benefits	US$ 13,813,524 (95% CI: −12,964,774–40,290,145)
PV Costs	US$ 3,241,685 (95% CI: 2,971,912–3,515,547)
NPV	US$ 10,564,828 (95% CI: −16,203,454–37,049,245)
BCR	4.25 (95% CI: −2.71–11.22)
IRR	91.38% (95% CI: 11.7–190.6%)

PV: present value; NPV: net present value; BCR: benefit–cost ratio; IRR: internal rate of return.

## Data Availability

All relevant data are within the paper.
